# Avoiding hyperoxemia during neonatal resuscitation: time to response of different SpO2 monitors

**DOI:** 10.1111/j.1651-2227.2010.02097.x

**Published:** 2011-04

**Authors:** Hernando Baquero, Ramiro Alviz, Armando Castillo, Fredy Neira, Augusto Sola

**Affiliations:** 1 Neonatology, Universidad del NorteBarranquilla, Atlántico, Colombia; 2 Neonatology, Medicina Alta Complejidad S.A.Barranquilla, Colombia; 3 Northeast Georgia Medical CenterGainesville, GA, USA; 4 Neonatology, President of IberoAmerican Society of Neonatology (SIBEN)

**Keywords:** Neonatal Resuscitation, Newborn, Oxygen saturation

## Abstract

**Aim:**

To assess the time to obtain reliable oxygen saturation readings by different pulse oximeters during neonatal resuscitation in the delivery room or NICU.

**Methods:**

Prospective study comparing three different pulse oximeters: Masimo Radical-7 compared simultaneously with Ohmeda Biox 3700 or with Nellcor N395, in newborn infants who required resuscitation. Members of the research team placed the sensors for each of the pulse oximeters being compared simultaneously, one sensor on each foot of the same baby. Care provided routinely, without interference by the research team. The time elapsed until a reliable SpO2 was obtained was recorded using a digital chronometer. Statistical comparisons included chi-square and student's *T*-test.

**Results:**

Thirty-two infants were enrolled; median gestational age 32 weeks. Seventeen paired measurements were made with the Radical-7 and Biox 3700; mean time to a stable reading was 20.2 ± 7 sec for the Radical-7 and 74.2 ± 12 sec for the Biox 3700 (p = 0.02). The Radical-7 and the N- 395 were paired on 15 infants; the times to obtain a stable reading were 20.9 ± 4 sec and 67.3 ± 12 sec, respectively (p = 0.03).

**Conclusion:**

The time to a reliable reading obtained simultaneously in neonatal critical situations differs by the type of the pulse oximeter used, being significantly faster with Masimo Signal Extraction Technology. This may permit for better adjustments of inspired oxygen, aiding in the prevention of damage caused by unnecessary exposure to high or low oxygen.

## Introduction

There have been numerous advances to improve newborn care during resuscitation including improved evaluation of heart rate and oxygen monitoring with aims to decrease the toxic effects of oxygen and to improve outcomes. Most of the investigations and reviews that have focused on these issues in the last 5 years are listed in the bibliography (1–17).

Despite these advances, oxygen is still used liberally during newborn resuscitation, in the delivery room and during NICU care (18,19), so many newborns are unnecessarily exposed to potentially damaging hyperoxia. It is therefore important to have accurate knowledge of the fraction of inspired oxygen used and of oxygen saturation levels during resuscitation in order to avoid hyperoxemia and oxidant stress in preterm and term infants (20,21).

Pulse oximetry is an important clinical tool for evaluating a patient's oxygenation status and guiding resuscitation (1,7,9,10,14,16) and is increasingly being used in the delivery room and during neonatal resuscitation. However, it has been known for over a decade that measurement failure rates can be high in critical conditions because of motion artefact and low perfusion that can lead to inaccurate readings, failures to report readings or freezing of displayed values (22–26). The time to obtain a reliable oxygen saturation reading during newborn resuscitation has been reported in a few publications (1,7,9) but technology has not been compared in detail in critical moments when infant motion and low perfusion are common and, therefore, it is not well known if there are performance differences between different types of pulse oximeters during those vital periods of time. The objective of this study is to assess if the time to obtain a reliable oxygen saturation reading differs by the type of pulse oximeter used during newborn resuscitation in unstable critical conditions.

## Material and Methods

This is a prospective observational study carried out in newborns who received resuscitation as standard of care either in the delivery room or in the NICU. The study took place in two centres (Clinica del Mar and Medicina Alta Complejidad S.A., in Barranquilla, Colombia), and it was approved by the IRB Committee of the institutions.

The inclusion criteria were newborn infants of any birth weight and gestational age who required partial or complete resuscitation in the delivery room or in the NICU. The sample for this study was by convenience, when all equipment was ready and the infant had a need for resuscitation. The only exclusion criteria were if the clinicians caring for the infant felt that the study could interfere with care or if the infant had previously placed intravenous or intra-arterial lines in one foot. The care provided was not altered by this investigation, and the research team members did not participate nor interfere with the care provided by the resuscitation team. By design, this study was not blinded because of the need for additional technology and expenses and to try to decrease interference with routine care as it occurs in emergency and unstable situations. For each of the subjects who met inclusion criteria, two different members of the research team simultaneously placed two pulse oximeter sensors, one for each of the two different pulse oximeters being compared. The monitors used in these centres routinely before this study were Nellcor and Ohmeda. One of the two monitors was placed on the hand as previously performed and was not removed form the study subject who was resuscitated in NICU. In the DR and in the NICU, the two monitors to be compared were placed in both feet (postductal region). The cable was connected to the pulse oximeters monitors first. The monitors were turned on, and alarms and sensitivity settings were set. In each study subject, the sensors were placed postductally, one on each foot, and then connected to the cable. They were placed in a standardized way, according to the directions for use provided by the manufacturer and as per routine clinical practice, using the highest sensitivity and two-second average readings.

The study did not interfere with routine clinical practice, and the research team was a different team than the one not providing care. Since the objective of this study was to compare two monitors placed at the same time on the same infant, the time to a reliable reading by each of the monitors was recorded by an observer of the research team, using a digital chronometer able to record different times simultaneously. The study, as designed, could not be masked to the researcher. Time zero for each pulse oximeter was the time when the sensor was adequately placed on the patient's foot and connected to the cable. A reliable reading was defined as the reading when the SpO2 monitor reported an oxygen saturation value with an adequate pulse rate signal. The study ended in each subject when the times for both monitors were completed and recorded, and care and monitoring continued to be provided independently by the care team, as it has been carried out until that time. The objective of this study was not to compare data with simultaneous blood gas analysis, and this was not included in the protocol submitted to IRB.

The SpO2 monitors used in this study included (i) Masimo Radical-7 SET (Masimo Corp, Irvine, CA) used in all subjects, (ii) Ohmeda Biox 3700 (GE Healthcare, United Kingdom) used on 17 subjects and (iii) Nellcor N395 monitor (Covidien, Boulder, CO). The sensors used in this study were the low noise optic probe neonatal sensor (LNOP) with the Radical-7, the OxiMax Max-N for the Nellcor and the E630 microfoam neonatal disposable sensor for Ohmeda. The main objective of the study was to compare the Radical-7 versus one of two other monitors simultaneously: (i) Ohmeda Biox and (ii) Nellcor N395, when placed in the same study subjects under the same unstable conditions. Based on anticipated time of response and in order to obtain a 40% difference in time (seconds), we calculated that the number of infants who needed to be recruited was 15 for each comparison. The statistical analysis was carried out using chi-square and student's *T*-test when appropriate. Statistical significance was considered when the p value was <0.05.

## Results

Thirty-six infants met inclusion criteria, and thirty-two were included in the study. The four not included had to do with concern of the clinicians, and sensors were not connected as planned.

Twenty-six of the 32 infants required resuscitation in the delivery room, and six infants were resuscitated in the NICU in acute unstable conditions. The median (interquartile range) gestational age was 32 (28–40) weeks, birth weight 1330 g (850–3220) and the Apgar at 5 min had a median score of five (2–6). Sixty-eight per cent of the infants were delivered by cesarian section.

Thirty-two paired measurements were made in the 32 infants. The Radical-7 was used on all 32 infants and paired with the Biox 3700 in 17 infants and with the Nellcor N-395 in 15 infants. The study ended in each subject in <6 min and care continued to be provided independently by the care team, as it has been until then. The majority of infants did not have blood gases obtained for clinical care during the duration of the study, and we elected not to compare data in the very few who had those results available.

The time in seconds to obtain a stable reading is shown in Table 1. Table 1A shows the mean, median and range of response times for the simultaneous measurements made for the Radical-7 and the Biox 3700, whereas Table 1B shows the mean, median and range of response times for the simultaneous measurements made for the Radical-7 and N-395. In subjects with both the Radical-7 and the Biox 3700, the mean (±SD) and median times for the Radical-7 to achieve a stable reading were 20.2 ± 6 and 20 sec, respectively, with a range of 18–26 sec, whereas the mean and median times for the Biox 3700 to achieve a stable reading were 74.2 ± 12 and 76 sec with a range of 38–98 sec. In subjects with both the Radical-7 and the N-395, the mean and median times for the Radical-7 to achieve a stable reading were 20.9 ± 4 and 21 sec with a range of 19–28 sec, whereas the mean and median times for the N-395 to achieve a stable reading were 67.3 ± 21 and 71 sec with a range of 40–90 sec. The response time differences between Radical-7 and the N395 and between the Radical-7 and the Biox 3700 were each statistically significant (p < 0.05). The tables also show that there was a trend of shorter time for the N-395 compared to Biox 3700 but the difference did not reach statistical significance. However, as mentioned in methods, the N-395 was not directly compared to Biox 3700 on the same study subjects.

**Table 1:** Time to achieve stable SpO_2_ reading. Comparison of simultaneously obtained data

Time (seconds)	Masimo (n = 17)	Ohmeda (n = 17)
A		
Mean ± SD	20.2 ± 6*	74.2 ± 12*
Median (extreme values)	20 (18-26) 76	(38-98)*
B		
	Masimo (n = 15)	Nellcor (n = 15)
Mean ± SD	20.9 ± 4**	67.3 ± 12**
Median (extreme values)	21 (19-28)	71 (40-90)**

* p = 0.02

** p = 0.03.

## Discussion

Based on the findings of this study, we can conclude that rapid and clinically useful readings of oxygen saturation can be obtained with pulse oximetry during neonatal resuscitation, but the time to obtain a stable saturation reading is dependent on the type of pulse oximeter selected.

It has been known for years that during the transition from intrauterine to neonatal life, the SpO2 is ‘low’ and varies from infant to infant in normal conditions, and it has been reported that SpO2 values of about 95% are reached at or after 10 min of life in normal infants (9,14,27,28).

Additionally, we have published about the relationship of SpO2 to PaO2 and have found that when SpO2 reads 85% to 93% there was almost never a hyperoxemic PaO2 (29). However, it is not known exactly yet what SpO2 targets and levels are safe during the resuscitation and intensive care of full term newborns and premature infants (30,31).

In 1989, Diab and Kiani invented a new type of pulse oximeter with Signal Extraction Technology (SET), a set of proprietary signal processing algorithms that make it possible to obtain more accurate oxygen saturation and pulse rate readings even during challenging conditions such as patient motion and low perfusion (25). Many studies have shown Masimo SET pulse oximetry to be more reliable and accurate than other pulse oximetry technologies in clinically unstable conditions such as in critically ill patients in the ICU, NICU, after cardiopulmonary bypass and cardiac surgery and in the paediatric postanaesthesia care unit (22–24). We chose to do two determinations at one time in the two feet in order to ensure that both were in same territory in relation to the ductus arteriosus (postductal) at a period of life and in conditions that the ductus could play a significant role in perfusion and readings. We clearly understand the potential differences between preductal and postductal saturations and that at times the determinations in the right wrist are obtained faster and are more reliably than in other sites. However, if we chose that site for one monitor, the other monitor could have been at a disadvantage. We designed the study with a homogeneous neonatal population and chose to make postductal pulse oximetry simultaneous comparisons between two monitors, in order to avoid potential advantages of one monitor compared to another. Additionally, it would be very hard, if not impossible, to place 3 monitors at once for simultaneous comparisons in emergency situations; moreover, one of the three would by necessity be measuring a different territory form the other two (preductal or postductal).

The aim of this concise study, with <6-min duration in unstable conditions, was to compare response times of SpO2 monitors. By design, we did not plan to evaluate in these circumstances SpO2 readings to simultaneously obtained PaO2 values; the sample size would need to be significantly larger to obtain valid conclusions. We have previously performed comparisons of SpO2 and PaO2 with a significantly larger sample size of infants receiving supplemental oxygen and found that with SpO2 between 85% and 93% both hypoxia and hyperoxia occurred very infrequently (29). Finally, in this study, each comparison for response time was carried out simultaneously on the same baby. Therefore, gestational ages, birth weights, criteria for considering the need of resuscitation, caregivers and treatment utilized were the same for each infant studied and would therefore not impact the time to obtain a reliable SpO2 in one monitor compared to another.

SpO_2_ technology has changed and continues to change. Even though there are different generations of sensors, we do not know of any manuscript that shows different response times comparing one sensor against the other with the same monitor and we are also unaware of a similar study to ours. A recent randomized crossover study was performed in *stable* infants (32), without motion or low perfusion. The authors compared 4 different monitors applied consecutively (not simultaneously) in a randomly allocated order. They report that the total time for data to display was about 25–26 sec, including sensor placement time. The sensors used were LNOP HiFi sensors for the Masimo monitors and OxiMax Max-N sensors for the Nellcor monitors; the Masimo sensors were applied and connected more quickly. The same sensors were used in the current study, which was performed in critical unstable conditions and with simultaneous and not consecutive measurements. An additional difference between the studies is that we defined time zero *after* sensor placement. The fastest reliable reading was in about 27 sec. If in unstable conditions the time for sensor placement was also shorter with Masimo SET as reported in stable conditions (32), the benefits in reliable readings compared to other monitors may even be larger than what we report here, but this needs to be evaluated. Furthermore, in that study (32) and in ours, no comparison is carried out about perfusion index, as no oxygen saturation monitor other than Masimo has this capability.

We and others (2,3,6,8,11,15,17,30) have shown that avoiding hyperoxia in the preterm and also in the term newborn is extremely important, because hyperoxia is associated with potentially serious side effects like retinopathy of prematurity, oxidant damage to the lungs and brain and others. Clinical guidance with accurate and prompt SpO2 monitoring is essential to avoid hyperoxia even for brief periods of time.

In summary, in this study, we found that there are significant differences in the response of pulse oximeters during neonatal resuscitation; the pulse oximeter with SET providing the fastest response time. The speed and reliability of this technology can be of help for clinicians to more accurately adjust the fraction of inspired oxygen during newborn resuscitations, thus preventing or minimizing damage secondary to unnecessary exposure of oxygen and hyperoxemia and to wide fluctuations in oxygen levels.
